# Global adoption of 6-month drug-resistant TB regimens: Projected uptake by 2026

**DOI:** 10.1371/journal.pone.0296448

**Published:** 2024-01-05

**Authors:** Aastha Gupta, Sandeep Juneja, Victor Babawale, Nurov Rustam Majidovich, Norbert Ndjeka, Phuong Thi Mai Nguyen, Parpieva Nargiza Nusratovna, David Robert Omanito, Tiffany Tiara Pakasi, Yana Terleeva, Atyrkul Toktogonova, Yasir Waheed, Zaw Myint, Zhao Yanlin, Suvanand Sahu

**Affiliations:** 1 TB Alliance, New York, NY, United States of America; 2 National Tuberculosis and Leprosy Control Programme, Abuja, Nigeria; 3 National TB Programme, Dushanbe, Tajikistan; 4 National Department of Health, Pretoria, South Africa; 5 National Tuberculosis Programme, Hanoi, Vietnam; 6 National TB Programme, Tashkent, Uzbekistan; 7 Disease Prevention and Control Bureau, Philippine Department of Health, Manila, Philippines; 8 National Tuberculosis Programme, Ministry of Health, Jakarta, Indonesia; 9 National Tuberculosis Programme, Kyiv, Ukraine; 10 National TB Centre, Bishkek, Kyrgyzstan; 11 National TB Control Programme, Islamabad, Pakistan; 12 National TB Programme, Naypyidaw, Myanmar; 13 National Center for TB Control and Prevention, National Tuberculosis Reference Laboratory of China, Chinese Center for Disease Control and Prevention, Beijing, China; 14 Stop TB Partnership, Geneva, Switzerland; Kings College Hospital, UNITED KINGDOM

## Abstract

**Background:**

The WHO has issued a call to action urging countries to accelerate the rollout of new WHO-recommended shorter all-oral treatment regimens for drug-resistant TB (DR-TB), which remains a public-health crisis. The all-oral, 6-month BPaL/M regimen comprises 3–4 drugs: pretomanid used in combination with bedaquiline and linezolid, with or without moxifloxacin. This regimen has been recommended by the WHO for use in DR-TB patients instead of ≥9-month (up to 24-month) regimens. This study aims to project this regimen’s use, along with its components bedaquiline, pretomanid and linezolid, and other treatments for DR-TB globally through 2026. It is intended to guide global health stakeholders in planning and budgeting for DR-TB interventions. Projected usage could help estimate cost of the individual components of DR-TB regimens over time.

**Methods:**

Semi-structured interviews were conducted with national TB programme participants in key countries to gather intelligence on established plans and targets for use of various DR-TB treatment regimens from 2023 to 2026. These data informed development of projections for the global use of regimens and drugs.

**Results:**

Consistent global growth in the use of shorter regimens in DR-TB treatment was shown: BPaLM reaching 126,792 patients, BPaL reaching 43,716 patients, and the 9-11-month all-oral bedaquiline-based regimen reaching 13,119 patients by 2026. By 2026, the longer all-oral regimen is projected to be used by 19,262 patients, and individualised treatment regimens by 15,344 patients.

**Conclusion:**

The study shows BPaL/M will be used in majority of DR-TB patients by 2024, reaching 78% by 2026. However, national efforts to scale-up, case-finding, monitoring, drug-susceptibility testing, and implementation of new treatments will be essential for ensuring they are accessible to all eligible patients in the coming years and goals for ending TB are met. There is an urgent need to engage communities in capacity building and demand generation.

## Introduction

The World Health Organization (WHO) is urging countries to facilitate access to fully oral treatment regimens for patients with drug-resistant tuberculosis (DR-TB) [1, 2]. Although some countries have accelerated their TB response, DR-TB remains a public health crisis. According to WHO estimates, 450 000 people fell ill with DR-TB in 2021, of whom about 191 000 died. A 2017 study that forecast increasing rates of multidrug-resistant (MDR) and extensively drug-resistant (XDR) TB in four countries with high DR-TB burden emphasised that additional efforts are required to reverse the epidemic of DR-TB [3]. Accelerating the diagnosis and treatment of DR-TB is a primary component of the WHO’s End TB Strategy and a key target in the political declarations of the 2018 and 2023 United Nations High-Level Meetings on TB [4, 5]. However, these goals are at risk because of implementation and funding challenges in enabling access to diagnosis and care. Only one in three people with DR-TB are currently being detected globally. Of those, just over half are treated successfully [1].

In its December 2022 update on treatment guidelines, the WHO recommended the use of a 6-month treatment regimen comprising the new drug pretomanid (developed by the non-profit TB Alliance) used in combination with bedaquiline and linezolid, with or without moxifloxacin (BPaLM and BPaL, respectively), rather than the 9-month or longer 18-month regimens in DR-TB patients. The rationale for this recommendation is based on: a) evidence from the TB-PRACTECAL trial, which showed much improved treatment success rates with the BPaL/M regimen (86 to 88%) of 6 months’ duration compared with current standard-of-care regimens (52%), as well as lower levels of treatment failure, death and loss to follow-up; and b) data from the ZeNix trial that demonstrated high success rates of 89% with BPaL, as seen in the Nix-TB trial, were maintained in pre-XDR-TB cases, with reduced linezolid dose of 600 mg and fewer adverse events [6–8]. Additionally, research has shown that patients find it easier to complete the shorter, all-oral DR-TB regimen than the longer regimens, which last up to 20 months [1].

Furthermore, shorter regimens cost less because of the reduction in both drug cost and associated care cost. As an example, the cost of the BPaL regimen was USD720 per treatment course at Stop-TB Partnership’s Global Drug Facility (Stop-TB/GDF) access prices until 2022 [9, 10]. This cost has been reduced to USD 564 per treatment by the volume guarantee facilitated by the TB Alliance between MedAccess and Viatris in December 2022 [11]. This has brought the price of the new regimens close to that of conventional 9- to 11-month treatments. However, when we add healthcare, logistical, distribution and indirect patient costs, the saving with the new regimen is considerably greater: 40–75% per patient depending on the country [9, 12].

The objective of this study was to project the use of BPaL/M and its components, bedaquiline, pretomanid and linezolid, in DR-TB treatment globally until the year 2026. This information would be important for global health stakeholders, including donors, policy makers, and manufacturers, in the planning of resources and prioritisation. The outputs of the study will also help determine future cost–volume correlation for the individual components of DR-TB regimens.

## Methods

In 13 countries accounting for 69% of DR-TB incidence in 2021, we conducted semi-structured interviews (S1 and S2 Tables) with national TB programme (NTP) staff and/or designated local experts (respondents) first in 2020 and then between August 2022 and January 2023 to gather intelligence on country targets and planned regimens for DR-TB treatment for the time horizon of 2023–2026. These countries are China, India, Indonesia, Kyrgyzstan, Myanmar, Nigeria, Pakistan, Philippines, South Africa, Tajikistan, Ukraine, Uzbekistan and Vietnam. The selection of countries was based on the DR-TB burden in the countries, and their willingness to share information. However, the information has been shared on condition of anonymity and the aggregate values are added in this paper. Questionnaires were circulated in advance, and respondents were given a chance to prepare before the interview. Follow-up interviews were held, or was information obtained by email if respondents could not provide complete responses during the interview. The interviewees consulted notification data from 2017–2021, on the basis of which they were able to project use in the next few years. We complemented the data with additional information from country national strategic plans and Global Fund grant requests where available. We have excluded the Russian Federation—which accounts for 8% of DR-TB incidence—from this analysis because of the current situation in the country and the uncertain timelines of regulatory approval for the new drug pretomanid. For the countries that account for the remaining 23% of DR-TB incidence in 2021, we assigned a percentage increase/decrease for patients on treatment as well as for the number of patients using each regimen that is equivalent to the average such increases/decreases across interviewed countries.

**Fig 1:**
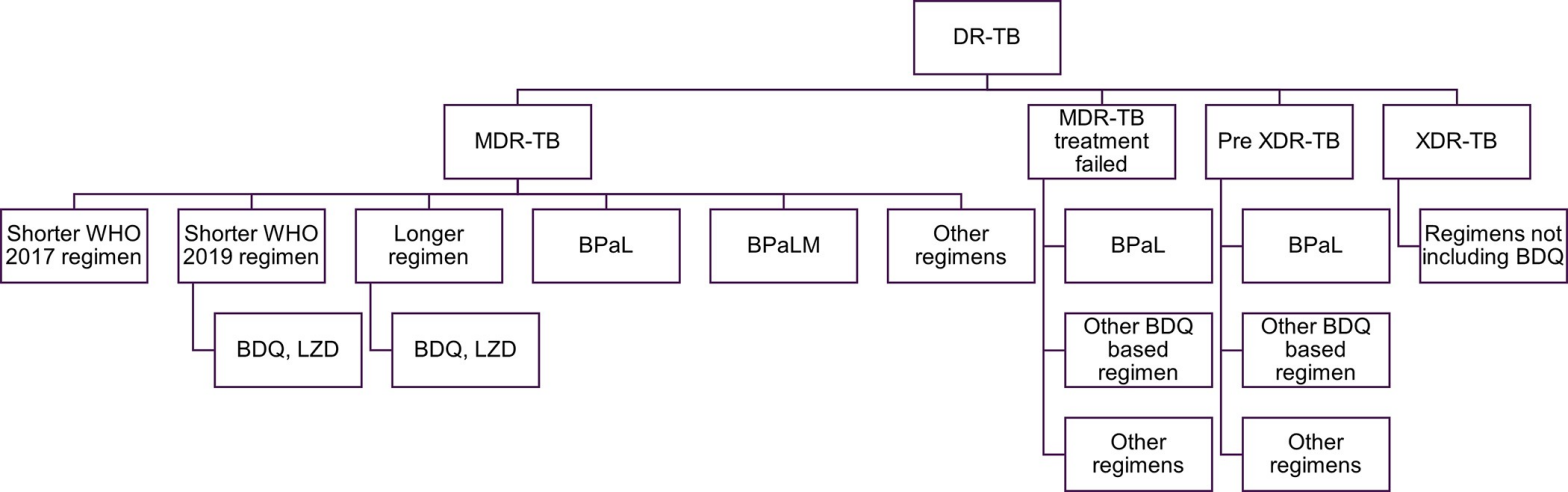
Allocations of regimens used by disease type.

To inform uptake forecasts, first, each country projected the number of patients in four categories: MDR-TB, MDR-TB treatment failed, pre-XDR-TB (MDR-TB with additional fluoroquinolone resistance), and XDR-TB (pre-XDR-TB with additional resistance to Group A drugs including bedaquiline and linezolid) (Fig 1). Then, eligibility of regimens or drugs was defined within these categories (Table 1). Lastly, within each of these four categories, the respondents allocated the number of patients to the respective drugs and regimens. The detailed analysis for this part of the study was limited to drugs used in the new DR-TB regimens: pretomanid, bedaquiline and linezolid.

**Table 1:** The four patient categories and drug regimens projected for each.

Patient category	Bedaquiline	Linezolid	BPaL	BPaLM
**MDR-TB**	X	X	X	X
**MDR-TB treatment failed**			X	
**Pre-XDR-TB**	X	X	X	
**XDR-TB**				

For MDR-TB patients, the respondents allocated the patients to a) the shorter regimen recommended by the WHO in 2016 (not containing bedaquiline or linezolid); b) the shorter all-oral bedaquiline-containing regimen recommended by the WHO in 2019 (containing bedaquiline, levofloxacin/moxifloxacin, ethionamide, ethambutol, isoniazid, pyrazinamide and clofazimine); c) individualised longer regimens (containing a mix of levofloxacin, bedaquiline, linezolid, clofazimine, cycloserine and other group C drugs); d) BPaL; e) BPaLM; or f) other individualised regimens [6, 13, 14].

For patients in the MDR-TB treatment failed and pre-XDR-TB groups, the respondents projected the percentage of patients likely to have initial MDR-TB treatment failure and then be re-treated in 2023‒26, as well as those on treatment for fluoroquinolone resistance. This was based on observed trends in their respective countries. With this patient base, the respondents allocated the percentage who could use a) BPaL; b) another bedaquiline-based regimen; or c) other individualised regimens. The number of such patients being re-treated with BPaL/bedaquiline declined over time based on each country’s speed in adopting BPaL regimens for initial MDR-TB treatment. However, for patients newly diagnosed with pre-XDR-TB, BPaL emerged as the regimen of choice over time, as in the WHO recommendations.

Based on the January 2021 definitions of XDR-TB, which defines patients resistant to Group A drugs such as bedaquiline and linezolid [15], there was no allocation of use of any bedaquiline- or linezolid-based regimens, including BPaL, for this patient group.

While projecting the uptake for new regimens, we discussed key considerations with the respondents and have defined timelines for completion of the operations research (OR) or conditional access programmes, where applicable in respective countries, and start of programmatic use in each country. For the programmatic uptake, we have considered need and time taken in countries for: a) national regulatory approval, if required (which varies by country and can take 6‒24 months); b) addition of the new regimens in national guidelines (which could be triggered either by inclusion in the WHO guidelines or successful results of the local OR); and c) availability of affordable generic drugs. We note that the last 2 years have been crucial for some countries to initiate the process of policy change, for which results of the ZeNix and TB-PRACTECAL trials as well as in experience from ORs, own or peers’, have been helpful. Additionally, the recent update of the WHO guidelines to include these regimens, along with the backing of the Global Fund in their grant proposals, has assisted countries in planning to move to newer regimens more quickly. However, some countries are experiencing procedural lag in developing national policy and guidelines and may expect to start using BPaL/M programmatically from late 2023/early 2024 onwards. Respondents have projected to start programmatic use of BPaL/M, keeping these considerations and timelines in mind for their own respective countries.

### Sensitivity/scenario analyses

We conducted two one-way sensitivity analyses to estimate change in usage of the new regimens with: a) every year delay or expedited programmatic use of BPaL/M in each country; and b) change in uptake of BPaL/M (±10% from projected country plans).

Additionally, we conducted a scenario analysis to estimate the use of pretomanid if all countries were to switch at least 85% of DR-TB patients to BPaLM for MDR-TB and BPaL for pre-XDR-TB starting in 2024, compared to the estimates of each country.

### Patient involvement

Patients were not involved in the study design, conduct or outcomes, as patient numbers were projected nationally based on interviews with national TB programme managers and staff only.

### Ethical statement

Request for ethical approval or informed consent was not deemed necessary as the data collected reflected national projections. All respondents agreed with the publication of these national level projections data in an aggregated, anonymised way.

## Results

The study projects a consistent global growth of number of patients treated for DR-TB from ~134 000 patients in 2021 to ~218 000 patients in 2026. Countries have cited two reasons for this increase. First, countries are aiming to return to pre-COVID treatment numbers and second, some countries have started national programmes to increase case finding and treatment. Of the 13 countries interviewed, four have estimated a more than two-fold increase in treatment numbers while another three have estimated a more than 50% increase.

**Fig 2:**
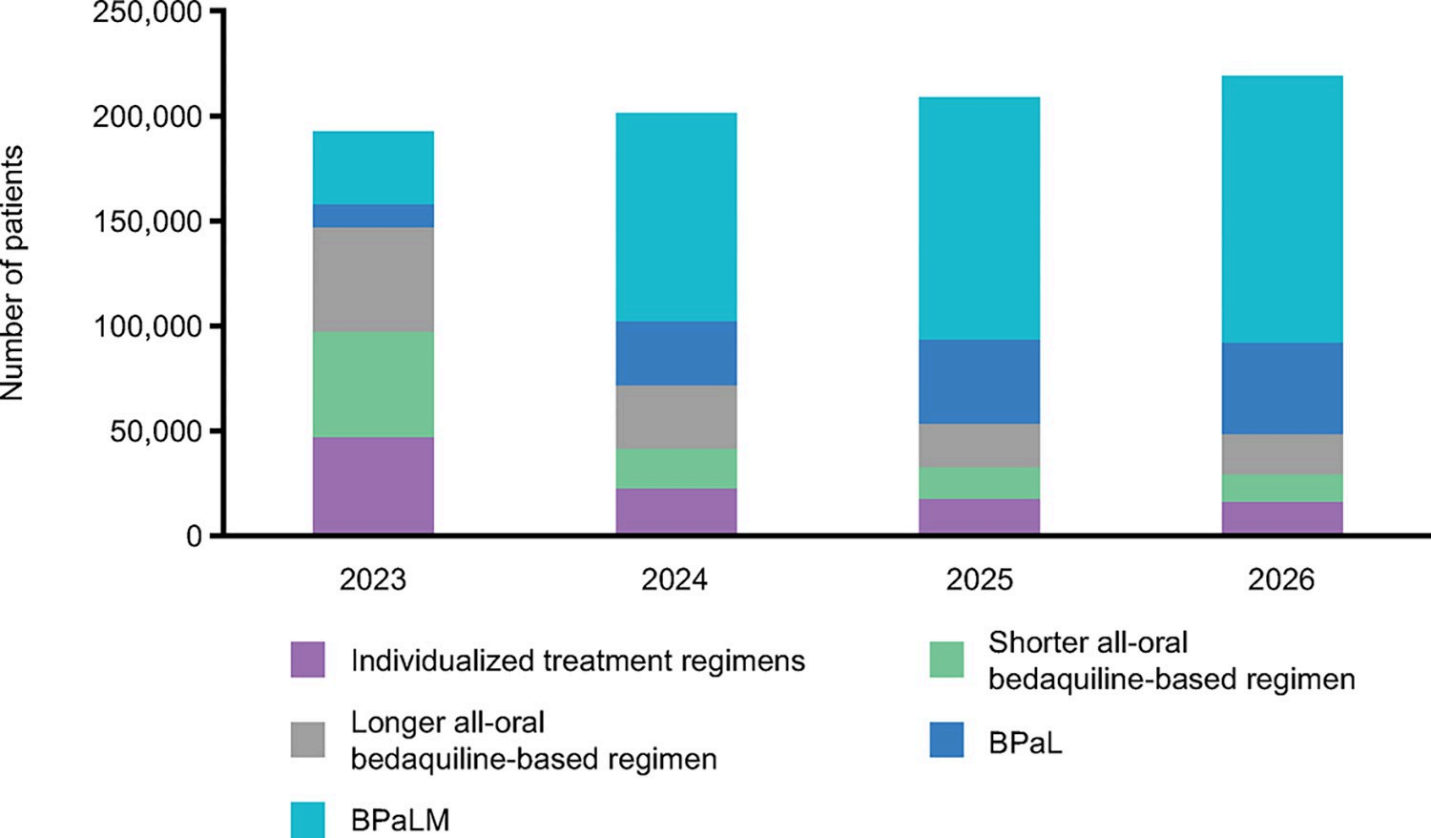
Projected use of regimens for MDR-TB globally between 2023 and 2026. BPaL: pretomanid + bedaquiline + linezolid; BPaLM: pretomanid + bedaquiline + linezolid + moxifloxacin; MDR-TB: multidrug-resistant TB.

The study projects consistent global growth in the use of shorter regimens: BPaLM reaching 126 792 patients, BPaL reaching 43 716 patients, and shorter 9-month all-oral bedaquiline-based regimen reaching 13 119 patients, in 2026. The longer regimen all-oral regimen is projected to be used by 19 262 patients by 2026 as are individualised treatment regimens at 15 344 (Fig 2). All above are annual numbers.

It is to be noted that the shorter regimen recommended by the WHO in 2016 is now phased out, to be replaced by the 9–11 months all-oral bedaquiline-containing regimen recommended by the WHO in 2019, referred to as the shorter all oral bedaquiline-based regimen.

It is interesting to note that while most countries are planning to shift to shorter regimens of either 6 months BPaL/M or 9–11 months all-oral bedaquiline-based regimen, a few—four of the 13 interviewed—are still estimated to have ≥25% of their MDR-TB patients using longer regimens by 2026. However, it is important to note that, in total, only about 9% of MDR-TB patients are estimated to be using the longer regimens globally by 2026. The remaining patients using the longer or individualised regimens are treatment failed, pre-XDR-TB or XDR-TB patients.

### Bedaquiline

**Fig 3:**
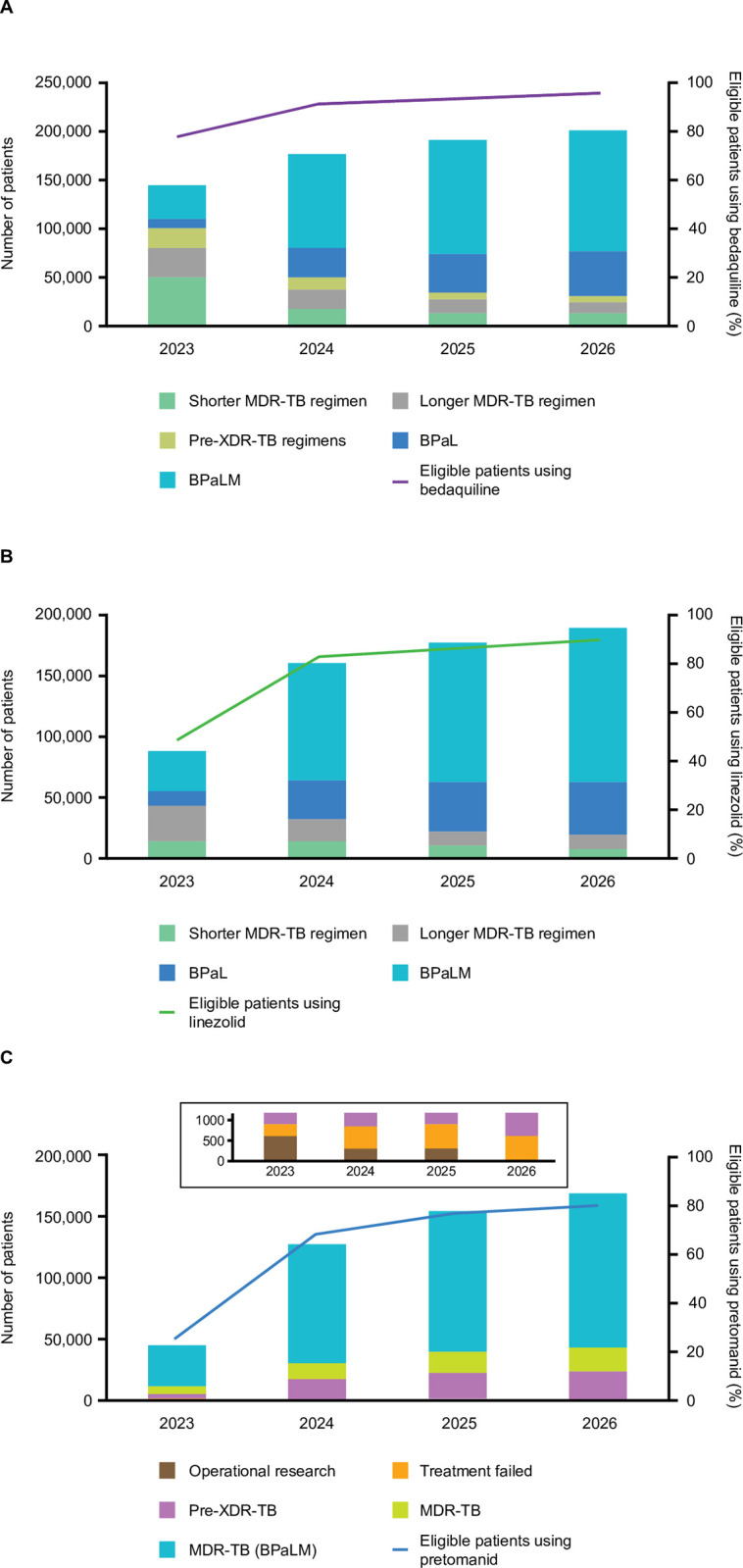
A. Projected use of bedaquiline. B. Projected use of linezolid. C. Projected use of pretomanid in various regimens globally between 2023 and 2026. Inset in C shows expansion of the primary y-axis to display the operational research and treatment failed data more clearly. BPaL: pretomanid + bedaquiline + linezolid; BPaLM: pretomanid + bedaquiline +linezolid + moxifloxacin; FQ: fluoroquinolones; MDR-TB: multidrug-resistant TB; XDR: extensively drug-resistant.

Projected use of bedaquiline would increase from 145 638 patients in 2023 to 202 920 patients by 2026, respectively representing 78% and 96% of the eligible patients estimated in 2023 and 2026 (Fig 3A). This increase is consistent with the trend of increased use of the drug reported in the WHO TB Report 2022 [16].

The two assumptions behind the increased use of bedaquiline are; firstly, all countries interviewed were planning to use bedaquiline in the longer and shorter regimens; however, for two of the countries, it was estimated that <50% of their patients on longer regimens would be using the drug. Secondly, all countries are progressing to shorter bedaquiline-containing regimens, including BPaL/M, over the period of this analysis, reaching a range of 70–100%, with one exception. Notably, 96% of all eligible patients are estimated to be using the drug by 2026.

### Linezolid

Projected use of linezolid would increase in the coming years to reach 188 503 patients by 2026, accounting for 89% of eligible patients (Fig 3B). The use of linezolid remains consistent in the range of 50–100% between 2023 and 2026 in the longer regimens. In the case of shorter regimens, while some countries are increasing the use of linezolid, others continue to use ethionamide instead. However, the use of linezolid shows an increase mainly from the greater use of BPaL/M.

### Pretomanid-based regimen (BPaL/M)

By December 2022, more than 50 countries had accessed pretomanid either through direct procurement or via Stop-TB Partnership/GDF to initiate the BPaL regimen (Viatris, unpublished data) [17], while others have planned procurement. This resulted in ~7000 patients accessing the new regimen cumulatively by the end of 2022. Programmatic use is estimated to result in more than 170 000 patients using pretomanid-based regimen by the year 2026 (Fig 3C).

Each country interviewed has planned to increase the use of BPaL/M as recommended by WHO [6], however, the pace of the implementation and adoption is different in each country. Of the total patients projected to use pretomanid by 2026 globally, 86% would be MDR-TB patients using BPaL/M, 13% would be pre-XDR-TB patients, and the remaining 1% would be those whose MDR-TB treatment failed. Globally, 83% of MDR-TB patients and 80% of all eligible patients are projected to use BPaL/M.

### Sensitivity/Scenario analyses

**Fig 4:**
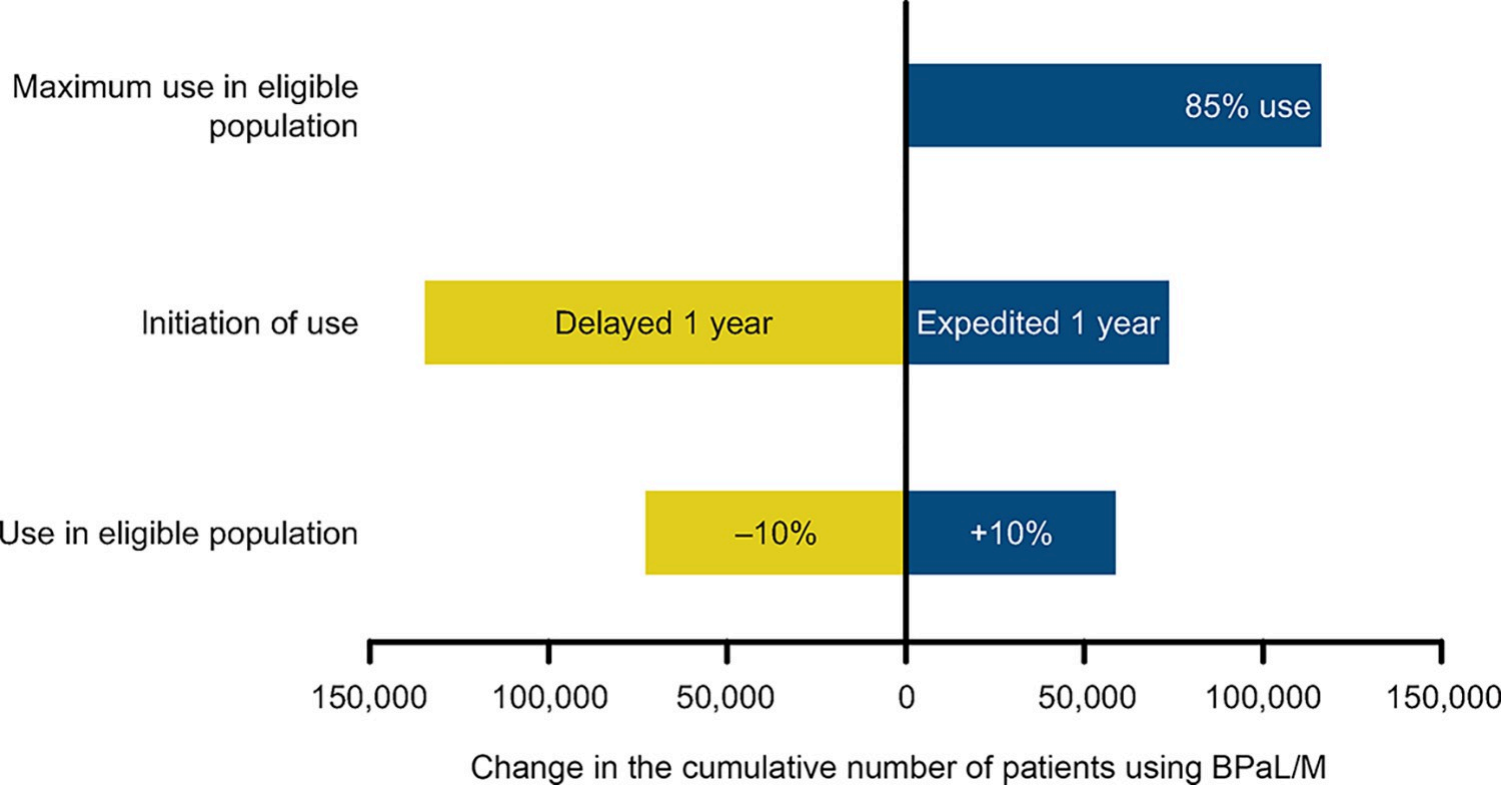
Sensitivity/scenario analyses of cumulative use of pretomanid-based regimens. BPaL: pretomanid + bedaquiline + linezolid; BPaLM: pretomanid + bedaquiline + linezolid + moxifloxacin.

In our one-way sensitivity analyses, delayed and expedited programmatic use of BPaL/M by 1 year resulted in a respective reduction and increase of 27% and 15% in cumulative use over the period 2023‒2026. Of the eligible population across patient categories, a change of +10% and –10% of patients provided with the BPaL/M regimen would result in an increase of 12% and a reduction of 15%, respectively, in cumulative use over the period 2023‒2026 (Fig 4, Table 2).

**Table 2:** Sensitivity/scenario analyses of cumulative use of pretomanid-based regimens.

Variable	Cumulative Baseline use of BPaL/M	Negative change	Positive change
**Use in eligible population (less 10% or increase by 10%)**	501,122	427,665 (-15%)	561,010 (+12%)
**Initiation of Use (1 year delayed by each country or 1 year earlier by each country)**	501,122	356,574 (-27%)	575,086 (+15%)
**Use in 85% of DR-TB population**	501,122		617,947 (+23%)

In the scenario analysis, if, instead of the numbers projected by the respondents, 85% of all MDR-TB and pre-XDR-TB patients were to use BPaLM and BPaL from 2024 onwards, it would lead to a 23% increase in the cumulative use of this regimen, from 501 122 to 617 947 patients, by 2026 (Fig 4, Table 2).

## Discussion

By aggregating the interviews from 13 countries with high DR-TB burden and adding average uptake for other countries, we projected an increase in the uptake of bedaquiline, linezolid and pretomanid (BPaL/M regimen) over the coming years.

By end of 2023, all countries interviewed intend to discontinue the injectable-containing shorter regimen for MDR-TB previously recommended by the WHO and transition most patients to all-oral bedaquiline-based regimens. The major driver of the increase in uptake of bedaquiline is its use in shorter regimens, including the BPaL/M regimen (Fig 3A). This implies that bedaquiline will continue to be a key drug in regimens for DR-TB.

The recent WHO recommendation of using 600 mg of linezolid daily has increased the acceptance of the drug, resulting in its much wider projected use compared with previous years. The recommendation and subsequent adoption of BPaL/M in countries from 2023 onwards also lead to the high use of linezolid.

While most patients are projected to be put on shorter regimens, about 9% of MDR-TB patients are estimated to be still using the longer regimens globally by 2026.

As with any new drug or regimen, programmatic uptake for pretomanid-based regimen is largely dependent on efforts to facilitate access to the drugs. The TB Alliance led LIFT-TB and access-oriented projects in other countries, as well as other efforts such as Stop-TB Partnership/TB REACH and USAID funded projects to provide technical assistance or accelerate commencement of OR in select countries, have sparked the uptake of pretomanid-containing novel regimens, starting with several pathfinder countries [18, 19]. To ensure speedy and affordable access to quality-assured pretomanid, the TB Alliance has thus far licensed the drug to its global commercialisation partner, Viatris, in addition to the generics manufacturers, Macleods, Hongqi Pharmaceuticals and Lupin. Since October 2019, the product had been available directly from Viatris worldwide and through Stop-TB Partnership/GDF in 150 countries at a price of USD 364 for a full 6-month treatment course [20]. This price has been further reduced to USD 240 by the volume guarantee facilitated by the TB Alliance between MedAccess and Viatris [11]. These efforts, along with the recently updated WHO guidelines, are expected to expedite the uptake of pretomanid. The transition period varies between countries and depends on each country’s acceptance of the updated WHO guidelines, local policy development, funding, training of clinical staff and health workers in the field, procurement of pretomanid for the new regimen, and, in some cases, liquidation of existing stocks of drugs. However, respondents in the interviews have indicated that the all-oral nature, short treatment duration, cost-effectiveness, and reported high efficacy of the BPaL/M regimen is expected to result in faster uptake than with other regimens in the past.

The same reasons have been cited by respondents for their projections of increased number of patients on treatment. It is projected that the ease of treatment and reduced cost will help the country programs increase the number of patients on treatment and reduce the gap between incidence and treatment.

It is noted in this study that the rate of drug susceptibility testing (DST) has been increasing over the last few years, from 9% to 26% and from 22% to 56% over 2015‒2018 for first- and second-line DST, respectively [21]. However, this is an area in which countries agreed they need urgent additional focus and investments. With wider usage of bedaquiline, there is an increased need for even greater availability of testing for that drug. Additionally, there is need for establishing break point for pretomanid, the newest MDR-TB drug and development of testing. Respondents projected an increase in the number of patients treated, estimating a likely increase in testing.

The COVID-19 pandemic has highlighted the limitations of TB care around the world. The response has disrupted TB services worldwide, particularly in countries with the highest burden, and reduced access to TB and MDR-TB diagnosis [22]. However, all countries interviewed intend to increase the number of notifications and treatments in the coming years to make up for the shortfall in 2020, as well as increase notifications and treatments further. As a result, the estimated number of DR-TB treatments will rise over the next few years.

Countries such as Indonesia, Kyrgyzstan, Myanmar, Nigeria, Pakistan, Philippines, South Africa, Tajikistan and Ukraine have already planned to start programmatic use of BPaL from late 2023/early 2024 onwards. It should be noted that these countries had the support from organizations such as TB Alliance and others, from WHO/TDR (the Special Programme for Research and Training in Tropical Diseases). Such early adopter countries would next need capacity building and community engagement to ensure smooth roll out throughout their respective territories. Countries that have not yet implemented BPaL/M could benefit from the experiences of the early adopter countries mentioned above to augment advocacy, capacity building, and training to ensure the process is minimally disruptive, rapid leverages existing tools to result in swift and efficient uptake of BPaL/M.

### Limitations

There are certain limitations to this study. We excluded Russia, which accounts for 8% of global incidence, from this analysis because of the current political situation and uncertainties of use of new regimens in the country. It is likely that regulatory approval of pretomanid and subsequent changes in policy to implement the drug may take longer in Russia than estimates achieved by global extrapolation. The countries included in this study accounted for 69% of global MDR-TB incidence; therefore, 23% of the projections were extrapolated to reach global estimates.

The projections are estimates based on each country’s assessment of the time taken to transition to newer regimens. While we have tried to capture these individually, the projections are subject to unanticipated changes such as delays in publishing national guidelines, delay in order placement, etc.

We have employed individual country data on use of drugs since 2019 to understand the uptake of new TB drugs. However, the lack of availability of Global Fund procurement data for TB drugs in the public domain has been a limiting factor in our understanding of the global market for new TB drugs.

## Conclusion

The study projects consistent global growth in the use of shorter regimens over the period 2023–2026: BPaLM reaching 126 792 patients (58% of DR-TB treatments), BPaL reaching 43 716 patients (20% of DR-TB treatments), and shorter all-oral bedaquiline-based regimen reaching 13 119 patients by 2026. The longer regimen all-oral regimen is projected to be used by 19 262 patients by 2026 as are individualised treatment regimens at 15 344.

The global health community will need to prepare for introduction of the pretomanid-based regimen as soon as possible and expand to include all eligible patients. This will involve alignment of key stakeholders, updating of national guidelines, scale-up of DST, patient engagement, education and training of clinicians, health workers, and affected communities on the safety and efficacy of such regimens, and procurement and distribution of the drugs. To this end, the WHO Global TB Programme has initiated the WHO BPaLM Accelerator Platform for regular discussion and coordination between stakeholders and countries to consider urgent and tangible actions and to ensure global coordination and dialogue between the main stakeholders.

This study will be informative for global health stakeholders, including donors, policy makers and manufacturers, by supporting planning and budgeting for DR-TB interventions. An estimation of future usage could help in volume-based pricing interventions for these key DR-TB medicines. Above all, national efforts to scale-up DST and implement new treatments will be essential for ensuring that these medicines are accessible to all eligible patients in the coming years.

## Supporting information

S1 Table Questionnaire for national tuberculosis program. (PDF)

S2 Table Questionnaire capturing country projections. (PDF)
