# Correction: Global adoption of 6-month drug-resistant TB regimens: Projected uptake by 2026

**DOI:** 10.1371/journal.pone.0298250

**Published:** 2024-01-31

**Authors:** The PLOS ONE Staff

## Notice of Republication

This article was republished on January 22, 2024, to correct error in the data availability statement: Personal email addresses of multiple personnel were erroneously provided in the data availability statement. An incorrect version of the S1 File was also published in error. The publisher apologises for these errors. Please download this article again to view the correct version.
