# Supplementary material for: Global adoption of 6-month drug-resistant TB regimens: Projected uptake by 2026
Source: PLoS One. 2024 Jan 5;19(1):e0296448. doi: 10.1371/journal.pone.0296448 (PMC10769048; doi:10.1371/journal.pone.0296448)
Supplement: S1 Table — (PDF) [file pone.0296448.s001.pdf]

Questionnaire for NTPs

1	**Regulatory Approval and Waiver**			
a	Is national regulatory approval a requirement for a product that is on WHO guidelines, is USFDA approved and listed on the Global Drug Facility to:			
	i. Start Operations Research	Yes	No	
	ii. Start programmatic use	Yes	No	
b	IF YES (to either of the above questions):<br>How long does it normally take for regulatory approval for a TB product from time of submission of dossier?	Months		
c	Would an MDR-TB or XDR-TB drug qualify for a waiver or an expedited regulatory review?	Yes	No	
	IF YES, what is the timeline?			
	- For waiver	Weeks		
	- For regulatory review	Weeks		
d	Any details on the pre-requisites and process?			
e	Do the following speed up the waiver process, and if so then by how much time:			
	i. Donation of product	Yes	No	Weeks
	ii. Research exceptions (such as: use in Operations Research)	Yes	No	Weeks

2	**Operational Research (OR)**				
a	Are you planning an OR or pilot for BPaL before programmatic use?	OR	Pilot	Neither	
	IF you are planning an OR or pilot:				
	i. Of the below, which patient populations will OR/pilot cover? And how many patients for each? How many patients do you expect to enrol in first 3 months and every 3 months going forward in each segment?	Total Number of Patients	First 3m	Next 3m	Next 3m
	- MDR-TB				
	- MDR Treatment Intolerant				
	- MDR Treatment Failed				
	- XDR-TB				
	- Pre-XDR TB				
	ii. What is the current status of the study? *(e.g., protocol being finalized, ethics committee approval awaited, regulatory approval awaited, etc)*				
	iii. When is the patient enrolment expected to be initiated? (please specify month/year or quarter/year)				
	iv. How long do you plan to continue enrolment?	<6 months	6-12 months	13- 18 months	
	vi. After enrolment, how long do you expect it will take for data analysis, compilation and reporting of results?	Weeks			
	vii. If the results of OR/pilot are satisfactory, will you automatically switch to programmatic use or wait for WHO Guidance to be updated?	Switch	Wait		
	IF AN AUTOMATIC SWITCH, will you:				
	- Wait for the entire OR/pilot to be over before taking a decision on programmatic use, *or*				
	- Be able to decide on programmatic use, based on trends visible from the study as it proceeds				
	viii. If you can switch to programmatic use during the study, how much patient data would you require?	Patients			
	ix. Can there be any reason other than lack of WHO guidelines for a delay between results of a favourable OR and decision to use the regimen programmatically? Please elaborate				
	x. Do national guidelines need to be updated before programmatic use?	Yes	No		
	IF YES:				
	- How long does the guideline update process take?	Months			
	- What will be the trigger for inclusion in national guidelines: Successful results from OR/pilot (interim or final) or WHO guidelines?	Results from OR/pilot	WHO guidelines		
b	IF you are not planning an OR/pilot for BPaL				
	i. Do you plan to start using BPaL programmatically:				
	- Without waiting for WHO guidelines	Yes	No		
	- Only after WHO guidelines are issued	Yes	No		
	- For some patients who don't have any feasible option per WHO guidelines	Yes	No		
	If yes, how many such patients do you estimate you will provide BPaL to?	Patients			
	ii Do you plan any other approach for access to BPaL? If so, please elaborate				

3	**Financing**		
a	Is funding secured for commencing OR/pilot for BPaL and later, introduction of the regimen programmatically?	Yes	No
	If Yes		
	i. Is the funding domestic or international?	Domestic	International
	ii. Is use of BPaL part of your National Strategic Plan?	Yes	No
	If No		
	How do you plan to move ahead with the OR for BPaL?		

4	**Bedaquiline and Linezolid Specific questions**							
a	When did the programmatic use of bedaquiline start? *(Note: Bedaquiline was first recommended by WHO in January 2013)*							
b	Number of patients using BDQ in the past 3 years as per GDF data is:	2016<br>Data provided	2017<br>Data provided	2018<br>Data provided	2019 (march)<br>Data provided			
	What are the target number of patients who would be using bedaquiline in the coming years for MDR-TB:	2019	2020	2021	2022	2023	2024	2025
	i. Within the BPaL regimen							
	ii. In the shorter MDR-TB regimen							
	iii. In the longer MDR-TB regimen							
c	Are you using linezolid in the shorter MDR-TB regimen?	Yes	No					
	- If YES, for how many months?	Months						
d	How many patients are/were using linezolid in your program in 2018, 2019 and 2020?	2018	2019	2020 (till May)				
	- In the longer MDR-TB regimen							
	- In the shorter MDR-TB regimen							
e	What are the target number of patients who would use linezolid in the coming years?	2021	2022	2023	2024	2025		
	- In the longer MDR-TB regimen							
	- In the shorter MDR-TB regimen							
f	For how many months do patients use linezolid in the longer MDR-TB regimen?	Months						

5	**Data, Estimations**							
A	***DS-TB Notifications***	2014	2015	2016	2017	2018		
i	DS-TB notifications in your country for the last few years as per WHO database is:	Data provided	Data provided	Data provided	Data provided	Data provided		
	Do you agree with these numbers?	Yes	No					
	DS-TB notifications in your country for the last few years as per national reports:							
		2019	2020	2021	2022	2023	2024	2025
ii	In your opinion (or as per Strategic Plan), what would be the number of DS-TB patients notified in the coming years?							
iii	In your opinion (or as per Strategic Plan), what would be the number of DS-TB patients treated in the coming years?							

B ***Drug Susceptibility Testing (DST)***

i Data from the WHO database for the last few years show:

	2014	2015	2016	2017	2018
Of the DS-TB notifications, the percentage of patients being tested for RR/MDR TB is:	Data provided	Data provided	Data provided	Data provided	Data provided
The number of patients with RR/MDR TB is:	Data provided	Data provided	Data provided	Data provided	Data provided
Of the tested patients, the <u>percentage</u> of positive patients being put on RR/MDR treatment is:	Data provided	Data provided	Data provided	Data provided	Data provided
The <u>number</u> of patients on treatment is:	Data provided	Data provided	Data provided	Data provided	Data provided

ii In your opinion, do you think these percentages will increase in the next few years?

Yes	No

DR TB notified patients as per national data :

	2019	2020	2021	2022	2023	2024	2025
iii In your opinion, how many patients would be confirmed MDR-TB in the coming years?							
iv In your opinion, what would be the percentage of notified DS-TB patients being tested for RR/MDR TB be in the coming years?							
v In your opinion, of all MDR/RR-TB patients, what would be the percentage (or number) of patients being put on treatment for MDR-TB?							
- Of these, how many (percentage or number) would be on shorter MDR-TB regimen recommended by WHO in 2017?							
- Of these, how many (percentage or number) would be on modified shorter MDR-TB regimen containing bedaquiline (WHO 2019)?							
- Of these, how many (percentage or number) would be on longer MDR-TB regimen?							

C ***MDR Treatment Failure***

	2014	2015	2016	2017
i The MDR-TB failure rate in your country for the last few years as per WHO database is:	Data provided	Data provided	Data provided	Data provided

ii Can you assume this rate (as a proportion to MDR-TB treated patients) to be constant in the next 3-5 years?

Yes	No

	2019	2020	2021	2022	2023	2024	2025
iii If no to (ii), how do you expect this rate to change over the coming years, or what will be safe assumption to make?							
iv What percentage of treatment-failed patients in your country can be switched to BPaL in your opinion? *(Note that MDR-TF is one of the approved indications for BPaL)*							

D ***MDR Treatment Intolerant***

i What is the current protocol for treating MDR-TB patients who are unable to tolerate their treatment?

	2016	2017	2018	2019
ii How many patients (%) treated for MDR TB are found to be intolerant to first line MDR drugs? *[WHO database does not report this data]*				

iii Can one assume this rate (as a proportion to MDR-TB treated patients) to be constant in the next 3-5 years?

Yes	No

	2021	2022	2023	2024	2025
iv If no to (iii), how do you expect this rate to change over the coming years, or what will be safe assumption to make?					
v What percentage of treatment intolerant patients will qualify for BPaL? *(Note that that MDR-TI is one of the approved indications for BPaL)*					

E ***Second Line Drug Susceptibility Testing***

i Data from the WHO database for the last few years show:

	2015	2016	2017	2018
Of the total MDR-TB laboratory confirmed patients, the percentage of patients being tested for susceptibility to second-line drugs is:	Data provided	Data provided	Data provided	Data provided
Of the tested patients, the <u>percentage</u> of positive patients being put on treatment is:	Data provided	Data provided	Data provided	Data provided
The <u>number</u> of these patients on treatment is:	Data provided	Data provided	Data provided	Data provided

ii In your opinion, do you think this percentage will increase in the next few years?

Yes	No

	2021	2022	2023	2024	2025
iii In your opinion, what would be the percentage of second line DST to lab-confirmed MDR patients in the coming years?					
iv In your opinion, of the tested patients, what would be the percentage (or number) of patients being put on treatment for XDR-TB and FQ resistance?					

v In the absence of Bedaquiline / linezolid DST how do you plan to design regimens containing these drugs as per WHO guidelines which recommend that patients should not have prior experience with these drugs of more than 2-4 weeks

F ***MDR-TB + Fluroquinolone Resistant***

	2017	2018
i Patients who have MDR-TB and are resistant to fluroquinolones in your country as per WHO database is:	Data provided	Data provided
Do you agree with these numbers? If not, please provide the correct numbers		
ii How many such patients have been on treatment?		

	2019	2020	2021	2022	2023	2024	2025
iii What is the target number of patients to be treated for FQ-resistant MDR-TB in the next 5 years?							
- Of these, how many do you estimate may use BPaL?							

G ***XDR-TB Treated Patients***

	2015	2016	2017	2018
i The number of lab-confirmed XDR-TB patients in your country for the last four years as per WHO database is:	Data provided	Data provided	Data provided	Data provided
Do you agree with these numbers? If not, please provide the correct numbers				
ii The number of patients treated for XDR-TB in your country for the last four years as per WHO database is:	Data provided	Data provided	Data provided	Data provided
Do you agree with these numbers? If not, please provide the correct numbers				

	2019	2020	2021	2022	2023	2024	2025
ii What is the target number of XDR-TB patients to be treated in the next 5 years?							
- Of these, how many do you estimate may use BPaL?							
