# Supplementary material for: Global adoption of 6-month drug-resistant TB regimens: Projected uptake by 2026
Source: PLoS One. 2024 Jan 5;19(1):e0296448. doi: 10.1371/journal.pone.0296448 (PMC10769048; doi:10.1371/journal.pone.0296448)
Supplement: S2 Table — (PDF) [file pone.0296448.s002.pdf]

COUNTRY PROJECTIONS

	2019	2020	2021	2022	2023	2024	2025	2026
**Total Patients on treatment (MDR+XDR+PreXDR)**								
**MDR-TB patients on treatment**								
**% of MDR-TB Patients on Regimens:**								
- short regimen WHO 2017								
- on modified short regimen 2019 (with BDQ)								
- on longer MDR-TB regimen								
- on BPaL								
- on BPaLM								
Total (100%)								
**MDR-TB patients on short regimen WHO 2017**								
**MDR-TB patients on modified short regimen 2019 (with BDQ)**								
BDQ Use (in shorter regimen WHO 2019)								
LZD Use (in shorter regimen WHO 2019)								
**MDR-TB patients on longer MDR-TB regimen**								
BDQ Use (in Longer regimen)								
LZD Use (in Longer regimen)								
**MDR-TB patients on BPaL**								
**MDR-TB patients on BPaLM**								
BPaL use in MDR-TB								
BPaLM use in MDR-TB								
BDQ use in MDR-TB								
LZD use in MDR-TB								
**MDR-TB patients failed treatment**								
BPAL Use (Treatment Failed)								
BPaL use in treatment failed patients								
**MDR-TB patients intolerant to treatment**								
**Pre-XDR TB treated**								
BPAL Use (Pre-XDR)								
BDQ Use (Pre-XDR) [other than BPaL]								
BPaL use in Pre-XDR patients								
BDQ use in Pre-XDR patients								
**XDR TB treated**								
BPAL Use (XDR)								
BDQ Use (XDR) [other than BPaL]								
BPaL use in XDR patients								
BDQ use in XDR patients								
BPaL Use in **Operational Research**								
**TOTAL BPaL Use**								
**TOTAL BPaLM Use**								
**TOTAL BDQ Use**								
**TOTAL LZD Use**								
